# Unlocking the potential of vitreous humor in biomarker discovery for Alzheimer's Disease and Alzheimer's Disease‐Related Dementias

**DOI:** 10.1002/alz70856_107504

**Published:** 2026-01-09

**Authors:** Dariusz Pytel, Brittany T Ivey, Dorea P Jenkins, Michelle Ackerman, Jennifer Belk, Carly Mitchell, Laura Davis, Mihyun Lim Waugh, Melissa A Moss, Chang Liu, Feng Ding, Jody F Longo, Steven L Carroll

**Affiliations:** ^1^ Medical University of South Carolina, Charleston, SC, USA; ^2^ University of South Carolina, Columbia, SC, USA; ^3^ University of Massachusetts Amherst, Amherst, MA, USA; ^4^ Clemson University, Clemson, SC, USA

## Abstract

**Background:**

Alzheimer's Disease (AD) and AD‐Related Dementias (ADRDs) represent a growing public health challenge, underscoring the urgent need for accessible and reliable biomarkers for early diagnosis, progression monitoring, and therapeutic response assessment. The search for reliable and accessible biomarkers remains a critical challenge in advancing early diagnosis and precision medicine. Vitreous humor (VH), a transparent ocular fluid in close anatomical and physiological connection with the central nervous system (CNS), represents a promising yet underutilized biofluid for biomarker discovery. Recent studies suggest that VH has potential as a proxy for brain neuropathology. We hypothesize that VH may also provide useful biomarker potential for other neurodegenerative diseases. We analyzed postmortem paired biosamples from VH, cerebral spinal fluid (CSF) and blood plasma from individuals with AD and other ADRDs to test this hypothesis.

**Method:**

Postmortem blood plasma, CSF, and VH samples (*n* = 133) were obtained from the Carroll A. Campbell, Jr. Neuropathology Laboratory at the Medical University of South Carolina. Samples were tested for Aβ40, Aβ42, GFAP, and NfL. Relative concentrations were measured using the Simoa Neurology 4‐Plex E (N4PE+) Advantage PLUS assay with a 4‐ fold dilution for blood plasma, 25‐fold dilution for VH and 400‐fold dilution for CSF on HD‐X analyzer (Quanterix, MA). All samples were processed per manufacturer's instructions in the immunoassay kits; we are currently analyzing the same samples for *p*‐Tau217. Artificial intelligence (AI) predictive modeling (including neural networks) using retrospective and prospective cohorts is being used to ensure accuracy and reliability.

**Result:**

Our findings reveal biomarker signatures (Aβ40, Aβ42, GFAP, and NfL) in the VH of AD and ADRDs. Moreover, we highlight the potential of these biomarkers for early disease detection and differentiation of AD from ADRD subtypes. We analyzed the relationship between VH biomarkers, blood plasma, CSF, clinical, and neuropathological measures.

**Conclusion:**

VH may be a valuable source for biomarker discovery in neurodegenerative disease such as AD, diffuse Lewy body disease and frontotemporal dementia (FTD). We anticipate that integration of VH and other biomarkers with AI‐driven data analysis will enhance diagnostic capabilities, ultimately supporting neurology and memory care clinicians in the future.

